# Evoked and Ongoing Pain-Like Behaviours in a Rat Model of Paclitaxel-Induced Peripheral Neuropathy

**DOI:** 10.1155/2018/8217613

**Published:** 2018-06-03

**Authors:** Lisa A. Griffiths, Natalie A. Duggett, Ann L. Pitcher, Sarah J. L. Flatters

**Affiliations:** Wolfson Centre for Age-Related Diseases, Institute of Psychiatry, Psychology and Neuroscience, King's College London, London SE1 1UL, UK

## Abstract

Paclitaxel-induced neuropathic pain is a major dose-limiting side effect of paclitaxel therapy. This study characterises a variety of rat behavioural responses induced by intermittent administration of clinically formulated paclitaxel. 2 mg/kg paclitaxel or equivalent vehicle was administered intraperitoneally on days 0, 2, 4, and 6 to adult male Sprague-Dawley rats. Evoked pain-like behaviours were assessed with von Frey filaments, acetone, or radiant heat application to plantar hind paws to ascertain mechanical, cold, or heat sensitivity, respectively. Motor coordination was evaluated using an accelerating RotaRod apparatus. Ongoing pain-like behaviour was assessed via spontaneous burrowing and nocturnal wheel running. Mechanical and cold hypersensitivity developed after a delayed onset, peaked approximately on day 28, and persisted for several months. Heat sensitivity and motor coordination were unaltered in paclitaxel-treated rats. Spontaneous burrowing behaviour and nocturnal wheel running were significantly impaired on day 28, but not on day 7, indicating ongoing pain-like behaviour, rather than acute drug toxicity. This study comprehensively characterises a rat model of paclitaxel-induced peripheral neuropathy, providing the first evidence for ongoing pain-like behaviour, which occurs in parallel with maximal mechanical/cold hypersensitivity. We hope that this new data improve the face validity of rat models to better reflect patient-reported pain symptoms, aiding translation of new treatments to the clinic.

## 1. Introduction

Paclitaxel is a highly effective anticancer agent with the major dose-limiting side effect of neuropathic pain. Patients describe an array of sensory symptoms in the hands and/or feet including numbness, tingling, ongoing pain, and mechanical and cold hypersensitivity. These symptoms significantly impact the quality of life, for example, pain on walking and inability to remove items from a fridge/freezer [1, 2]. Deep tendon reflexes and temperature and vibration perception may also be lost in severe cases. Mild weakness has been reported in some patients receiving very high doses of paclitaxel. However, paclitaxel-induced neuropathy usually presents as a predominantly sensory neuropathy, increasing in severity with higher cumulative doses, which persists for months to years after termination of treatment [3–7]. Meta-analysis of clinical studies indicates that some degree of neuropathy is reported in 44%–98% of paclitaxel-treated patients [8]. Alleviation or prevention of chemotherapy-induced neuropathic pain is a challenging clinical issue as many analgesics are ineffective (reviewed in [9, 10]), and only duloxetine has a moderate recommendation [9, 11].

Given the lack of treatment options for chemotherapy-induced neuropathic pain, it is important to understand the underlying mechanisms of this disorder. Using rat models can provide a means to investigate paclitaxel-induced neuropathic pain, thus enabling mechanistic studies that would be infeasible/unethical in humans. Several rodent models have been developed utilising different dosing regimens of paclitaxel (reviewed in [12]). These studies report the assessment of evoked pain-like behaviours using mechanical and thermal stimuli applied to the hind paw. However, the complete time course and resolution of these evoked pain-like behaviours are underreported.

Accurate modelling of ongoing pain in rodent models is challenging as patient reporting relies on verbal report. To better model patient symptoms, current pain-like behavioural techniques could be improved with the addition of ethological testing such as burrowing and spontaneous wheel-running assays. For rodents, burrowing is a natural behaviour that can be disrupted if normal physiology is altered, for example, by anxiety [13]. Previous studies have shown that burrowing behaviour is impaired in inflammatory and neuropathic pain models and can be reversed by analgesics at clinically relevant doses [14–16]. Spontaneous wheel running has also been shown to be an elective, natural behaviour [17]. Spontaneous wheel running is also impaired in inflammatory pain models [18, 19] and can be ameliorated by NSAIDs at doses devoid of antiallodynic effects [18]. These studies show that ethological testing can measure ongoing pain-like behaviours in rat models of chronic pain.

Here, we have used a rat model of paclitaxel-induced painful neuropathy evoked by intermittent systemic administration of low doses of clinically formulated paclitaxel to replicate treatment cycles. This paclitaxel dosing regimen has been frequently used by our lab [20–22] and other groups. However, our aims with this study were to more fully characterise the behavioural phenotype in this model without variation in the rodent strain, experimenter, drug formulation, or dosage administered, to describe the complete time course of evoked pain-like behaviours and motor coordination in this low-dose paclitaxel model, and to assess two ethologically relevant behavioural tests: spontaneous burrowing and nocturnal wheel running. Therefore, we report, for the first time, the evoked and ongoing pain-like behaviours associated with paclitaxel-induced neuropathy in rats.

## 2. Materials and Methods

### 2.1. Animals

Adult male Sprague-Dawley rats (175–200 g; Harlan/Envigo) were housed in groups of three or four in a temperature-controlled room with a 12-hour light/dark cycle. A phased “sunset” and “sunrise” lighting period over ∼30 min preceded complete dark/light periods. By 7 am, the room was fully illuminated. All cages contained sawdust bedding with environmental enrichment materials, and food/water was freely available. All procedures were conducted in strict accordance with the UK Animals (Scientific Procedures) Act 1986 and IASP ethical guidelines [23]. Protocols used were approved by the Ethics Review Panel of King's College London and conducted under the UK Home Office project licenses 70/6673 and 70/8015. Unless otherwise stated below, the rats were habituated to the testing environment for 20–30 minutes on three separate occasions before baseline testing. The rats were alert, not grooming or sleeping, during assessment of all evoked pain-like behaviours. Unless otherwise stated, behavioural assessment occurred between 8 and 11 am. Separate cohorts of rats were used for the assessment of the different behavioural tests to avoid the impact of different hind paw stimuli on other behavioural assessments. The only exception to this was in one cohort of rats where cold hypersensitivity was assessed in the morning and motor coordination in the afternoon. A total of 109 rats were used in these studies. An equally sized concurrent vehicle-treated group was tested in parallel with the paclitaxel-treated rats in all experiments. No rats were excluded from these studies due to weight loss or their response to behavioural testing.

### 2.2. Administration of Paclitaxel

Clinically formulated paclitaxel solution for infusion (6 mg/ml; CP Pharmaceuticals UK or Actavis UK) was diluted with 0.9% sterile saline (Fresenius Kabi, UK) to achieve a 2 mg/ml solution for injection. To replicate the clinical formulation, a vehicle stock solution was made using 1 : 1 solution of Cremophor EL (Sigma, UK) and ethanol. When using paclitaxel from Actavis, the vehicle stock was supplemented with 2 mg/mL sodium citrate (Sigma, UK). Prior to administration, one part of vehicle stock solution was diluted with two parts of 0.9% sterile saline. The rats were dosed with 2 mg/kg paclitaxel or equivalent volume of vehicle solution intraperitoneally on four alternate days (0, 2, 4, and 6). Animals were dosed according to their weight (1 ml/kg) and were immediately returned to their home cages. All paclitaxel/vehicle injections were administered in the early afternoon 1–3 pm. Both paclitaxel and vehicle solutions are clear solutions with the same viscosity, which enables blinding procedures (see below).

### 2.3. Mechanical Hypersensitivity

Mechanical hypersensitivity was assessed using von Frey filaments (Touch-Test™ Sensory Evaluators, Linton Instrumentation, UK) as previously described [20–22, 24]. The rats were placed in elevated, clear Perspex boxes (15 cm × 16 cm × 21 cm) with a wire-rung floor and allowed to acclimatise. When still alert, and with all four paws in contact with the floor, von Frey filaments (4 g, 8 g, and 15 g) were applied to the hind paws in the ascending order of force. Each von Frey filament was applied to five discrete points on the midplantar region of each hind paw and held in place for five seconds, and the number of withdrawals was recorded. All rats were tested using one von Frey filament on one hind paw before beginning to test the other hind paw with the same bending force filament. The scores of both hind paws were added together to give a withdrawal score out of ten for each von Frey filament. After habituation, three baseline measurements were taken on separate days prior to administration of paclitaxel/vehicle. von Frey withdrawal responses were then typically assessed on days 7, 14, 21, 28, 35, 49, 62, 77, 90, 104, 119, 132, 148, 166, and 174 to determine the time course of paclitaxel-induced mechanical hypersensitivity. After day 174, the rats were tested every 7–10 days until resolution of mechanical hypersensitivity. Resolution of mechanical hypersensitivity was variable between rats and was considered resolved when withdrawal responses had returned to baseline response levels on two separate occasions (spaced approx. 7 days apart). Data are combined from experiments with five small cohorts of rats (*n*=3–6 per treatment group), which were performed over a 2.5-year period. Over this 2.5-year period, there were unexpected changes in the colony of Sprague-Dawley rats and the brand of paclitaxel available from our regular suppliers. Therefore, to ensure consistency, additional cohorts were run by the same experimenter (LAG). Importantly, an equally sized concurrent vehicle-treated group was tested in parallel within each cohort at each time point. There is variability in *n* at different time points because it was not possible to test each cohort on all the same days. Sample sizes for each treatment group at each time point were as follows: *n*=21 at baseline and on days 7, 21, 28, 77, 90, 104, 132, 148, and 166; *n*=18 on days 62 and 119; and *n*=15 on days 14 and 35. As this combined data set is not uniform, it does not meet the requirements for a repeated measures ANOVA. Therefore, unpaired *t*-tests were used, and the Bonferroni correction was applied to account for repeated testing (see Section 2.10).

### 2.4. Cold Hypersensitivity

Cold hypersensitivity was assessed using acetone as previously described [24, 25]. The testing apparatus for cold hypersensitivity assessment was the same as used for mechanical hypersensitivity. 50 *µ*l of acetone was applied to the plantar surface of each hind paw using a P200 pipette and a stopwatch started. Care was taken to avoid the spread of acetone onto any of the animals' fur (this alters or masks the hind paw response as they will attend to the wetted fur only). Responses were graded as follows: 0 = no response; 1 = single withdrawal (lifting, flicking, or stamping the paw); 2 = multiple or prolonged withdrawals; and 3 = paw withdrawal and licked on the plantar surface. Responses were scored for 20 seconds following acetone application. If the rat did respond within this period, the rat's response was assessed for an additional 20 seconds. Previous extensive observations showed that if a rat had not responded within the first 20 seconds following acetone application, then no response would be observed in any additional time. The additional 20 seconds following an initial response allowed complete observation of the interrupted behaviour evoked by plantar hind paw acetone application. Responses were assessed for the left hind paw of each rat, followed by the right hind paw, and repeated three times. The scores from each hind paw response were added together to give a score out of a maximum of 18 points (six acetone applications multiplied by 3 points). Hind paw testing was nonconsecutive, with each rat tested on one hind paw before returning to test the first rat's other hind paw. A minimum period of 10 minutes had passed before the next acetone application was applied to the same hind paw. Data are combined from experiments with two cohorts of rats (*n*=6 per treatment group), which were performed over a 2-year period. Over this time, there were unexpected changes in the colony of Sprague-Dawley rats and the brand of paclitaxel available from our regular suppliers. Therefore, to ensure consistency, two cohorts were run by the same experimenter (LAG), resulting in a combined data set with *n*=12 per treatment group.

### 2.5. Heat Hypersensitivity

Heat hypersensitivity was evaluated using a radiant heat source [26]. The testing apparatus consisted of six clear Perspex boxes (dimensions 22 cm × 17 cm × 14 cm) with a glass floor. The plantar heel surface of each hind paw was stimulated with an infrared beam (Ugo Basile, Italy), which cut off when the rat withdrew its paw. The latency from initiation of stimulation to paw withdrawal was then recorded. Responses were assessed for the left hind paw of each rat, followed by the right hind paw, and repeated three times. Approximately eight minutes passed between stimulating the same hind paw. Latencies were added together to give the mean of six measurements. The infrared intensity was maintained at 149–162 mW/cm^3^ as measured using the Heat-Flux Radiometer throughout the time course investigated (Ugo Basile, Italy) (*n*=6 per treatment group).

### 2.6. Motor Coordination

Motor coordination was assessed using an accelerating RotaRod apparatus (Ugo Basile, Italy) [27]. The rats were habituated to the testing apparatus by placing their cages next to the apparatus with the motor switched on for ∼10 minutes on two occasions. The rats were placed on the apparatus, one to each lane, with an initial speed of 4 rpm. Once the rats were in position, the acceleration program gradually increased the speed of rotation from 4 to 40 rpm over five minutes (300 seconds). The latency time in seconds to fall onto the sensor platform was recorded. Two to four training sessions were given to ensure that all rats could stay on the apparatus for a minimum of 180 seconds, followed by one baseline measurement before commencing paclitaxel or vehicle treatment (*n*=6 per treatment group).

### 2.7. Burrowing Behaviour

Burrowing behaviour was investigated using custom-made burrowing tubes (with supplies purchased from B&Q plc, UK) in a protocol based on previously published studies [13, 15, 28, 29]. Plastic burrowing tubes were 32 cm in length and 10 cm in diameter and had one end sealed. The open end was raised by approximately 6 cm. Test cages contained an empty burrowing tube on sawdust bedding, with food/water ad libitum. Test cages were kept within the room where the rats were normally housed. The rats were housed in pairs and habituated to the test cages for 24 hours prior to the first training session. Training sessions consisted of the cage mates moving to a test cage (they had been habituated to) with the burrowing tube filled with 2500 g of 5 mm pea shingle (Porton Garden, Aquatics & Pets Centre, UK). Animals were trained in pairs for 2 hours, from 4 to 6 pm, after which they were returned to their original home cage with sawdust and environmental enrichment materials, without the burrowing tube. The following day, the process was repeated for the second training session. If any pairs of rats did not burrow in the first training session, the pairings were swapped with the rats that did show burrowing activity. For baseline testing, which occurred 24 hours following the second training session, the rats were placed individually in cages containing burrowing tubes filled with 2500 g of pea shingle at 4 pm. The rats were observed to record the time taken until burrowing was initiated (latency to burrow). After two hours, the amount of gravel left in the tube was measured to determine the amount of gravel burrowed. All animals burrowed more than 500 g at baseline, a previously described minimum requirement for study inclusion [15, 30]. In between subsequent burrowing sessions, the rats were housed with their original respective cage mate. The rats were assessed again on days 7 and 28 for the latency time to burrow and the amount of gravel burrowed. Burrowing sessions always took place at the same time each day (4–6 pm). The weight of gravel dislodged at baseline was used to group match the animals prior to drug administration, ensuring similar baseline burrowing behaviour between treatment groups. No animals were excluded from the analysis (*n*=6 per treatment group).

### 2.8. Spontaneous Wheel Running

Spontaneous wheel-running activity was recorded using Activity Wheels designed for rats (model BIO-ACTIVW-R; Bioseb, Boulogne, France). Software enabled recording of activity within a cage similar to the rat's home cage with dimensions of 48 × 31.5 × 47 cm and wheel dimensions of 34 cm height and 7 cm width. Animals had free access to food, water, and the wheel at all times. Activity wheel cages contained sawdust bedding only (no environmental enrichment materials) and were kept within the room where the rats were normally housed. Animals were individually housed in activity wheel cages during recording periods, after which they were returned to their home cage with the same cage mates. Animals were habituated to the activity wheel cage for one hour during the day (10-11 am), prior to overnight recordings. For overnight recordings, animals were placed in their activity wheel cage at 6 pm and were returned to their home cage at ∼9 am the following day. Previous studies have shown that the majority of spontaneous wheel running occurs during the night in rats [31], so the period was the focus in our study. Wheel-running data were split into the dusk phase (6-7 pm) and night phase (7.10 pm–6.20 am). The wheels were connected to a laptop with software that automatically recorded several parameters throughout the recording period: time spent on the wheel (active time), total distance travelled, maximal acceleration, the number of times the rat entered the wheel (access count), and mean speed. Prior to paclitaxel/vehicle administration, the rats were grouped based on matching-pairing of overnight total distances recorded at the baseline time point. Animals were placed in their activity wheel cage again on days 7, 28, and 35 for one hour during the light cycle to habituate and then from 6 pm to 9 am the following day. Animals were exposed to the same wheel and cage throughout all experiments. Due to limited equipment, two different cohorts of animals consisting of *n*=5 per treatment group were tested. Unexpectedly, during the first experiment, one animal receiving paclitaxel died during the injection period. We suspect that this was due to a subclinical infection in this animal, which was then exacerbated by paclitaxel administration. This is a highly unusual event, which has not occurred previously in our lab. As this experiment resulted in *n*=4 for the paclitaxel group and *n*=5 for the vehicle group, too low for robust statistical analysis, we repeated the experiment again. Therefore, the combined data set from both blinded experiments was *n*=9 for the paclitaxel group and *n*=10 for the vehicle group. No animals were excluded from data analysis based on their wheel-running behaviour. Baseline data were variable between animals, and this was taken into account in the statistical analysis by using ANCOVA as opposed to ANOVA.

### 2.9. Blinding and Randomisation

All behavioural testing was performed by a single experimenter, under blind conditions, to avoid unconscious bias. Animals were designated to experimental groups based on their respective baseline responses to each behavioural test. Behavioural extraneous variables were minimised by testing concurrent vehicle-treated groups of equal size throughout all experiments. Drug treatments were randomised within a given cohort of animals. Prior to drug/vehicle administration, injection vials were anonymised by a third party. At the end of the experiment, the identity of the treatment each rat received was revealed for data analysis.

### 2.10. Statistical Analysis

All statistical analyses were carried out using GraphPad Prism 6.07 or IBM SPSS Statistics 23. Typically, the statistical significance was accepted at *p* ≤ 0.05, with no further distinction made for *p* < 0.01. Weight gain in paclitaxel- versus vehicle-treated rats was assessed using two-way repeated measures analysis of variance (ANOVA). Mechanical hypersensitivity in paclitaxel-treated rats compared to concurrent vehicle-treated rats was analysed using two-tailed unpaired *t*-tests with Bonferroni correction for multiple comparisons. For example, on day 35 (the fifth repeated test from baseline), the significance was accepted if *p* < 0.01 (0.05 divided by 5); on day 119 (the eleventh repeated test from baseline), the significance was accepted if *p* < 0.0045 (0.05 divided by 11). To assess cold hypersensitivity, responses are categorised, and therefore, the nonparametric Friedman test (nonparametric equivalent to RM ANOVA) with Dunn's post hoc test was utilised, comparing the baseline scores in each group. The effect of paclitaxel on heat hypersensitivity and motor coordination was analysed using two-way repeated measures ANOVA. For burrowing behaviour, the gravel displaced and latency to initiate burrowing were analysed using one-way repeated measures ANOVA with Dunnett's post hoc test comparing the baseline measurements. The effect of paclitaxel administration on spontaneous wheel-running behaviour was analysed using two-way repeated measures ANCOVA (analysis of covariance), using baseline values as the covariate. Pairwise analysis was also conducted in SPSS, applying Bonferroni adjustment. No animals were excluded from any analyses conducted.

## 3. Results

**Figure 1:**
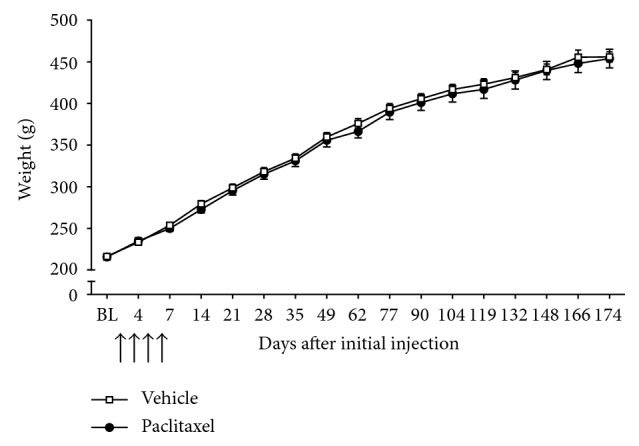
Time course of weight gain in rats following paclitaxel or vehicle administration. Graph shows mean ± SEM of rats' weight (g) from before paclitaxel/vehicle administration (BL) up to day 174. Arrows indicate four injections of 2 mg/kg paclitaxel or equivalent volume of vehicle on days 0, 2, 4, and 6 (*n*=18 per group).

All animals were healthy throughout the studies, without evidence of alopecia or diarrhoea. Paclitaxel- and vehicle-treated rats gained weight similarly throughout the studies, with no significant difference between paclitaxel- and vehicle-treated rats at any time point investigated (Figure 1; *n*=18 per group).

**Figure 2:**
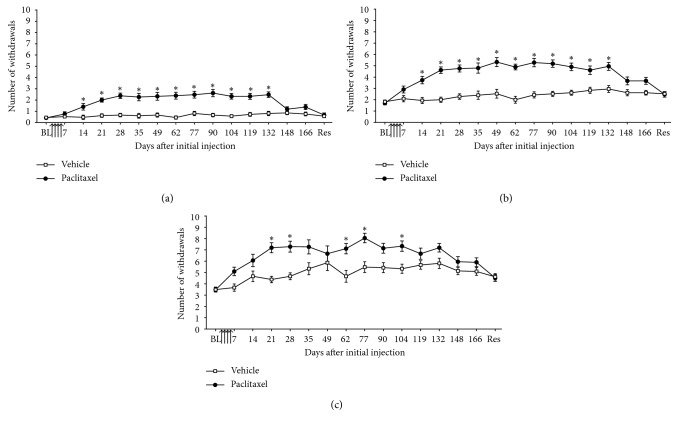
Time course of paclitaxel-induced mechanical hypersensitivity. Graphs show the mean ± SEM of the number of withdrawal responses to (a) von Frey 4 g, (b) von Frey 8 g, and (c) von Frey 15 g, respectively. Arrows indicate four injections of 2 mg/kg paclitaxel or equivalent volume of vehicle on days 0, 2, 4, and 6. BL = baseline before paclitaxel/vehicle administration. Res = resolution of mechanical hypersensitivity, which occurred between days 174 and 219. ^*∗*^*p* < 0.05, two-tailed unpaired *t*-tests with Bonferroni correction (*n*=15–21 per group).

Figure 2 shows that administration of low-dose paclitaxel induced a robust and long-lasting mechanical hypersensitivity. Following four systemic injections of 2 mg/kg paclitaxel on days 0, 2, 4, and 6, there was a short delay until the onset of mechanical hypersensitivity. On day 14, statistically significant mechanical hypersensitivity started to emerge, which peaked by day 28. Paclitaxel-induced mechanical hypersensitivity was significantly persistent with a long-lasting plateau, apparent for four months following paclitaxel administration (Figure 2; *n*=15–21; *p* < 0.05, two-tailed unpaired *t*-tests with Bonferroni correction). The resolution of paclitaxel-induced mechanical hypersensitivity was variable between different rats, resolving between days 174 and 219 (Figure 2). Mechanical hypersensitivity was considered to have resolved in an individual rat when withdrawal responses had returned to that rat's baseline responses before paclitaxel administration on two separate occasions (which were spaced approx. one week apart).

**Figure 3:**
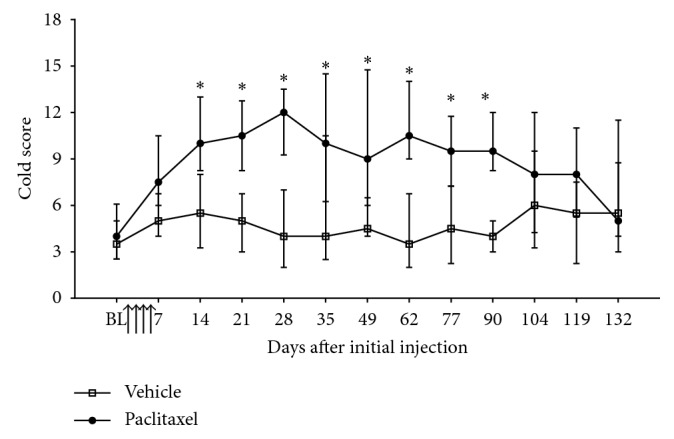
Time course of paclitaxel-induced cold hypersensitivity. Graph shows the median ± interquartile range of the hind paw response to acetone application catergorised by the cold score. BL = baseline before paclitaxel/vehicle administration. Arrows indicate four injections of 2 mg/kg paclitaxel or equivalent volume of vehicle on days 0, 2, 4, and 6. ^*∗*^*p* < 0.05, the Friedman test with Dunn's post hoc test comparing the baseline scores in each group (*n*=12 per group). As this analysis is nonparametric, the error bars appear larger than expected from parametric analysis.

**Figure 4:**
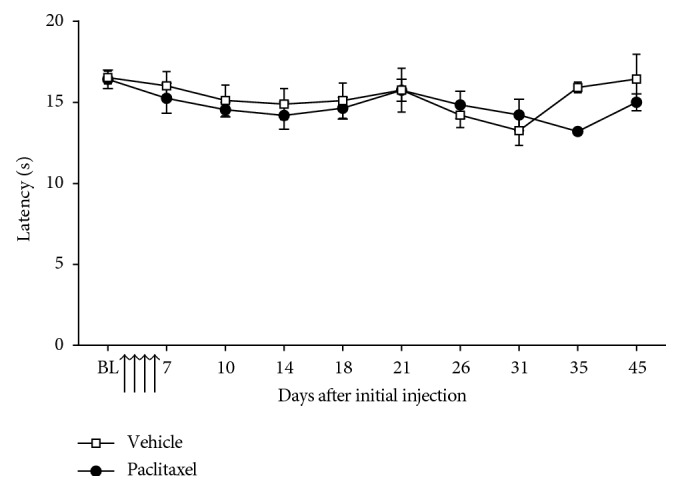
Effect of paclitaxel administration on heat hypersensitivity. Graph shows the mean ± SEM of the hind paw withdrawal latency to a radiant heat stimulus. BL = baseline before paclitaxel/vehicle administration. Arrows indicate four injections of 2 mg/kg paclitaxel or equivalent volume of vehicle on days 0, 2, 4, and 6 (*n*=6 per group).

Paclitaxel administration evoked a prolonged cold hypersensitivity, which was also delayed in its onset (Figure 3). Significant cold hypersensitivity was evident on day 14, peaked on day 28, and then persisted until day 90 following paclitaxel initiation (Figure 3; *n*=12 per group; *p* < 0.05, the Friedman test with Dunn's post hoc test comparing baseline scores). Following three consecutive nonsignificant data points, the cold hypersensitivity was deemed to have resolved on day 132 (Figure 3). Heat hypersensitivity was assessed until day 45 following paclitaxel initiation (Figure 4). We did not find evidence that paclitaxel evoked either early- or late-phase heat hypersensitivity compared to concurrent vehicle-treated controls (Figure 4; *n*=6 per group).

**Figure 5:**
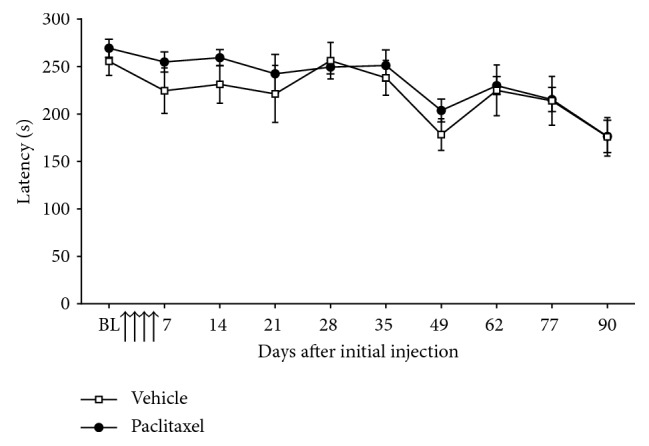
Effect of paclitaxel administration on motor coordination. Graph shows the mean ± SEM of the latency to fall from an accelerating RotaRod apparatus. BL = baseline before paclitaxel/vehicle administration. Arrows indicate four injections of 2 mg/kg paclitaxel or equivalent volume of vehicle on days 0, 2, 4, and 6 (*n*=6 per group).

As shown in Figure 5, low-dose paclitaxel administration did not significantly affect motor coordination assessed via latency to fall from an accelerating RotaRod apparatus. Motor coordination was assessed until day 90 following paclitaxel administration, and no change in latency was observed compared to concurrent vehicle-treated control animals (Figure 5; *n*=6 per group). The decrease in latency observed in both paclitaxel- and vehicle-treated rats at later time points of the time course is likely due to the increased size of the rats.

**Figure 6:**
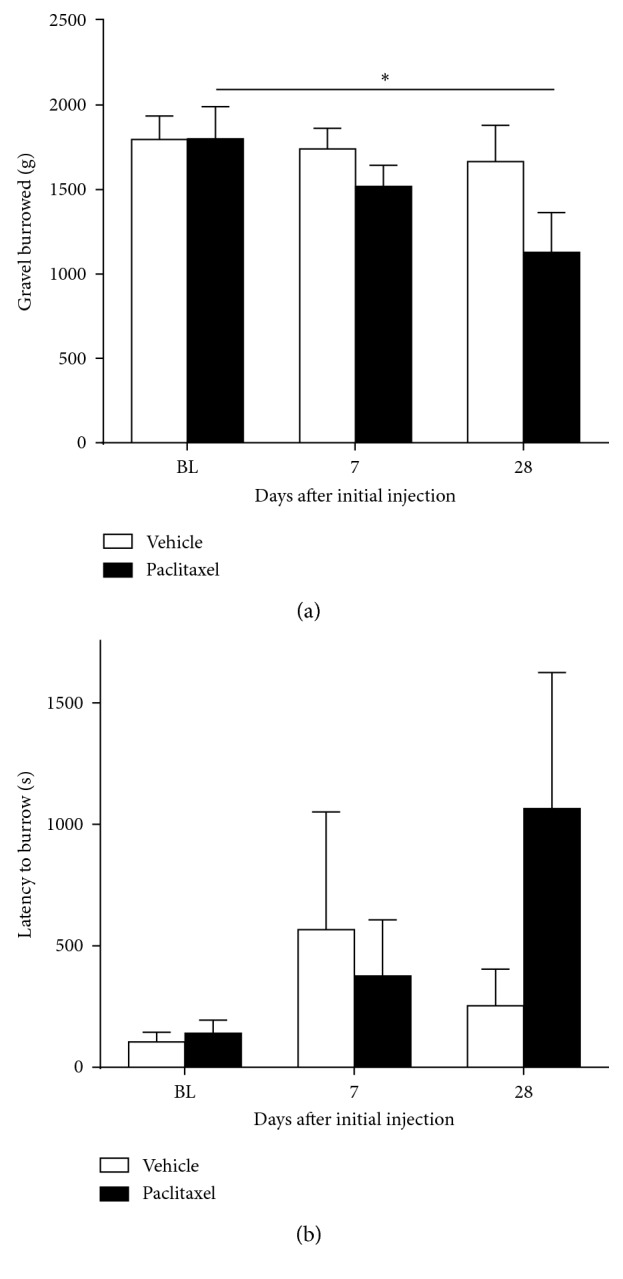
Effect of paclitaxel administration on spontaneous burrowing behaviour. Graphs show the mean ± SEM of (a) gravel displaced from burrowing tubes and (b) latency to start burrowing by individual rats before paclitaxel/vehicle administration (BL) and then on day 7 and day 28 following the initial injection of 2 mg/kg paclitaxel or vehicle. ^*∗*^*p* < 0.05, one-way repeated measures ANOVA with Dunnett's post hoc test comparing the baseline burrowing capacity (*n*=6 per group).

Figure 6 shows the effect of low-dose paclitaxel administration on spontaneous burrowing behaviour. On day 7, 24 hours after the last injection of paclitaxel, there was no statistically significant difference in the burrowing behaviour of paclitaxel-treated rats compared to vehicle-treated rats. However, on day 28 (the peak of mechanical and cold hypersensitivity), there was a significant 39% reduction in the total gravel burrowed by paclitaxel-treated rats compared to their baseline responses (Figure 6(a); *n*=6 per group; *p* < 0.05, one-way repeated measures ANOVA with Dunnett's post hoc test). No significant change in burrowing behaviour was observed in the vehicle-treated rats. There was a tendency of paclitaxel-treated rats to take a longer time to start burrowing on day 28 (Figure 6(b)), but this was not statistically significant.

**Figure 7:**
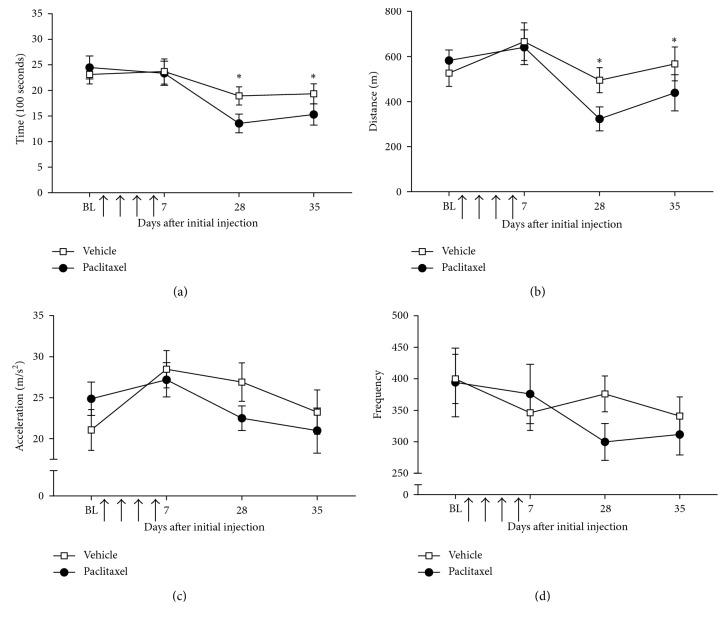
Effect of paclitaxel administration on overnight spontaneous wheel-running behaviour. Graphs show the mean ± SEM of the (a) time spent on the wheel, (b) total distance travelled, (c) maximal acceleration generated, and (d) number of times the wheel was accessed by individual rats overnight (7.10 pm–6.30 am) before paclitaxel/vehicle administration (BL) and then on day 7, day 28, and day 35 following the initial injection of 2 mg/kg paclitaxel or vehicle. Arrows indicate four injections of 2 mg/kg paclitaxel or equivalent volume of vehicle on days 0, 2, 4, and 6. ^*∗*^*p* < 0.05, two-way repeated measures ANCOVA with Bonferroni pairwise analysis (*n*=10 vehicle rats and *n*=9 paclitaxel rats).

Figure 7 shows the overnight (7.10 pm–6.20 am) spontaneous wheel-running behaviour of paclitaxel- and vehicle-treated rats on days 7, 28, and 35 after treatment initiation. On day 7, there was no significant difference between treatment groups on any of the parameters recorded. In contrast, on day 28, paclitaxel-treated rats showed a significant decrease in the time spent on the wheel (Figure 7(a); *p*=0.004) and total distance ran on the wheel (Figure 7(b); *p*=0.004) compared to concurrent vehicle-treated rats (*n*=9‐10 per group; *p* < 0.05, two-way repeated measures ANCOVA with Bonferroni pairwise analysis). On day 28, decreases were also observed in maximal acceleration (Figure 7(c); *p*=0.060) and the number of times they entered the wheel (Figure 7(d); *p*=0.054); however, these observations did not reach statistical significance. On day 35, paclitaxel-treated rats spent significantly less time on the wheel (Figure 7(a); *p*=0.038), with decreased total distance ran overnight (Figure 7(b); *p*=0.050) compared to concurrent vehicle-treated rats (Figures 7(a) and 7(b); *n*=9‐10 per group; *p* < 0.05, two-way repeated measures ANCOVA with Bonferroni pairwise analysis). There was no change in the mean speed between treatment groups at any time point investigated. Dusk-phase (6-7 pm) spontaneous wheel-running behaviour was also significantly impaired in paclitaxel-treated rats, but only on day 28 with two measures. Paclitaxel-treated rats spent significantly less time on the wheel (a 27.6% decrease) and ran less (a 32.1% decrease) during the dusk phase, compared to concurrent vehicle-treated rats (data not shown). These data suggest that monitoring activity during the dark phase provides the maximum information on impaired rat activity.

Further assessment of burrowing behaviour and spontaneous wheel running at later time points was not assessed because larger rats are unable to fit in the burrowing tubes and wheels. In addition, our RotaRod data (Figure 5) suggest that as they gained weight, they became less active. Therefore, the animals in these experiments were terminated after the time points shown in Figures 6 and 7.

## 4. Discussion

In this study, we have shown the complete time course of evoked pain-like behaviours following low-dose systemic paclitaxel administration. Administration of 2 mg/kg clinically formulated paclitaxel on days 0, 2, 4, and 6 induced a robust and persistent hind paw hypersensitivity to mechanical and cold stimuli. However, paclitaxel-treated rats did not develop heat hypersensitivity nor did they show any alterations in motor coordination. We also report, for the first time, evidence for ongoing pain-like behaviour in this low-dose paclitaxel model using two ethologically relevant behavioural tests. Paclitaxel-treated rats showed a reduction in both burrowing behaviour and nocturnal spontaneous wheel-running activity. Interestingly, we found that the time courses of evoked and ongoing pain-like behaviours were similar, with a delayed onset.

We consistently observed good overall health in paclitaxel-treated rats across different cohorts, with normal weight gain and no signs of alopecia or diarrhoea. Mild weight loss has been previously reported at this dosage [32], and significant weight loss has been observed with higher paclitaxel dosing regimens [33, 34]. Paclitaxel did not immediately evoke mechanical and cold hypersensitivity after administration but rather was delayed in its onset until a week after the last injection of paclitaxel. By day 14, significant mechanical/cold hypersensitivity was observed, which then increased to a peak approximately one month after the first paclitaxel injection. Paclitaxel-induced mechanical and cold hypersensitivity then persisted for several months. The decline of these behaviours differed, with cold hypersensitivity being completely resolved approximately 50 days earlier than mechanical hypersensitivity. Mechanical and cold hypersensitivity has also been described in paclitaxel-treated patients [3, 35]. The delayed and persistent hypersensitivity observed in this rat model is similar to the coasting phenomenon and persistence of pain seen in the clinic.

Rats in this investigation did not show any alteration in heat sensitivity following paclitaxel administration, compared to vehicle-treated controls, at any time point investigated. A previous study has also reported an absence of hind paw heat hypersensitivity following a cumulative dose of 8 mg/kg paclitaxel dissolved in 10% saline and Cremophor vehicle [36]. Other rodent models also report an absence of altered heat sensitivity following paclitaxel administration [33, 37]. Patients who report paclitaxel-induced pain show thermal hypoalgesia/increased heat detection thresholds [3, 4, 38]. However, this is observed only in animal models that use high cumulative doses of paclitaxel (25 mg/kg [32]; 32 and 80 mg/kg [34]; and 64.8 mg/kg [39]). It is possible that the marked neurodegeneration associated with high doses of paclitaxel may underlie the change in heat perception seen by others. This contrasts with the lack of change in heat perception seen in this model, which corresponds to an absence of marked neurodegeneration following the 8 mg/kg dosing regimen [40].

Motor symptoms are not commonly reported in patients receiving paclitaxel, unless they received high cumulative doses which evoked mild, distal weakness [3, 4, 41, 42]. We found that motor coordination was unaltered at any time point following paclitaxel administration. These findings confirm and temporally extend previous data showing that motor coordination was unaffected up to 3 weeks following similar or lower paclitaxel doses [36]. However, impairment of motor coordination assessed by RotaRod has been reported in rats following high cumulative doses of paclitaxel (25 mg/kg [32] and 36 mg/kg [43]).

More recently, there has been an emphasis on the requirement of better reporting of behavioural models and of developing methods to report ongoing pain [44–46]. Ethologically relevant behavioural assessment is a valuable methodology to implement as it measures normal rat behaviour/activity and thus can be an indicator of ongoing pain-like behaviour. In this study, we have shown that both burrowing behaviour and nocturnal spontaneous wheel running are impaired by low-dose paclitaxel administration. Importantly, this study provides the first evidence for ongoing pain-like behaviour in a rat model of paclitaxel-induced peripheral neuropathy. Burrowing behaviour was impaired on day 28 after paclitaxel initiation, but not immediately following paclitaxel dosing on day 7. Similarly, several measures of nocturnal spontaneous wheel running were significantly impeded in paclitaxel-treated rats on days 28 and 35 after paclitaxel initiation, but were unaffected on day 7, which is 24 hours after the last injection of paclitaxel. Therefore, the impairment of spontaneous ethological behaviours temporally reflects the occurrence of maximal evoked mechanical and cold hypersensitivity.

Alterations in burrowing behaviour have been reported in several rodent pain models evoked by peripheral nerve injury [15, 16, 47], hind paw inflammation [15, 16, 29], joint inflammation [30, 48], and antiretroviral drug treatment [28]. A recent investigation reported a standard burrowing protocol across multiple centres showing a consistent deficit in burrowing behaviour following CFA-induced hind paw inflammation [29]. This study also identified several variables which had a significant impact on sensitivity, including the number of training sessions provided and animal weight at the time of injury. Collectively, these studies suggest that burrowing can identify ongoing pain-like behaviour in several different pain models and may be confounded by several of the same factors which produce variability in evoked-pain testing.

Nocturnal spontaneous wheel running is perhaps a more novel behavioural assessment in research but reflects another ethological elective behaviour in rodents [17]. The ability to monitor this behaviour without the need for an experimenter to be present enables the possibility of long-term nocturnal assessment, as we report here. Spontaneous wheel running has not yet been used as widely as burrowing in preclinical pain studies. However, impairment of spontaneous wheel-running measures has been reported following hind paw inflammation in rats [19] and mice [18] and in a mouse model of osteoarthritis [49]. In our study, on day 28, we found that more measures (time spent on wheel, total distance ran, maximal acceleration, and wheel entries) of spontaneous wheel running were impaired during assessment overnight (7.10 pm–6.20 am) compared to assessment during the dusk phase (6-7 pm) only. This indicates an advantage to nocturnal assessment of this behaviour, when rats are most active [31].

It is unlikely that the impairment of spontaneous ethological behaviours by paclitaxel reported here is due to a deficit in motor function because paclitaxel-treated rats performed similarly to vehicle-treated rats on an accelerated RotaRod apparatus throughout the time course. We suggest that this reduction in spontaneous wheel-running and burrowing performance emulates the ongoing nature of pain reported by patients treated with paclitaxel. Furthermore, as there was no significant impairment of these spontaneous ethological behaviours on day 7, 24 hours after the last injection of paclitaxel, these behaviours are not a measure of acute drug toxicity.

## 5. Conclusions

Low-dose intermittent administration of paclitaxel induces long-lasting mechanical and cold hypersensitivity, without deficits in heat sensation and motor coordination. Paclitaxel also induces ongoing pain-like behaviour shown through the impairment of innate burrowing and wheel-running behaviours, which occurs in parallel with the peak of evoked pain-like behaviours. This study improves the face validity of this rat model of paclitaxel-induced peripheral neuropathy by detailing the measurable evoked and ongoing pain-like behaviours evoked by clinically formulated paclitaxel. The assessment of both evoked and ongoing pain-like behaviours may aid the translation of novel treatments to alleviate paclitaxel-induced neuropathic pain in the clinic.

## Data Availability

The data underlying this study are not suitable for data mining. However, the data are available from the corresponding author upon request or at the following lab's website: www.kcl.ac.uk/flatterslab.
